# Editorial: Iowa Gambling Task, Somatic Marker Hypothesis, and Neuroeconomics: Rationality and Emotion in Decision Under Uncertainty

**DOI:** 10.3389/fpsyg.2022.848603

**Published:** 2022-05-19

**Authors:** Ching-Hung Lin, Jong-Tsun Huang, Yao-Chu Chiu

**Affiliations:** ^1^Department of Psychology, Kaohsiung Medical University, Kaohsiung, Taiwan; ^2^Research Center for Nonlinear Analysis and Optimization, Kaohsiung Medical University, Kaohsiung, Taiwan; ^3^Graduate Institute of Biomedical Sciences, China Medical University, Taichung, Taiwan; ^4^Department of Psychology, Soochow University, Taipei, Taiwan

**Keywords:** Iowa Gambling Task, somatic marker hypothesis, neuroeconomics, rationality, emotion, decision-making, uncertainty

## Two Anthologies on the Study of Decision-Making Under Uncertainty

This anthology is the second in a series of *Frontiers in Psychology* Research Topics exploring how emotion and rationality interact in decision-making in an uncertain environment. The first anthology, “Twenty Years after the Iowa Gambling Task: Rationality, Emotion, and Decision-Making,” comprised 24 papers published separately between August 2012 and December 2015 in *Frontiers in Psychology* (Huang et al., 2018). These 24 articles covered the evolution of the Iowa Gambling Task (IGT) over two decades and included a variety of reviews, theoretical integration, clinical examinations, brain-imaging techniques, and model building, revealing numerous applications of IGT in studies of uncertain decision-making.

While the first anthology shed some light on the current state of IGT applications in decision-making, it raised further issues requiring illumination. These questions include the following: (1) What types of neurological, neuropsychological, and psychiatric dysfunction can be measured by IGT? (2) Can IGT bind to skin conductance responses (SCRs) to represent the critical paradigm of somatic markers? (3) What kind of implicit or explicit learning/decision-making ability does the IGT actually test? (4) Does expected value or gain/loss frequency predominantly influence selection behavior in the IGT? (5) Is it possible to develop a relatively powerful data-analysis scheme that can co-register relatively precise neural responses to specific choice behaviors exhibited in the IGT, such as events of winning, losing, and the switch pattern that occurs when different cards are selected (Chiu et al., 2018)?

To re-examine these fundamental issues generated after the first anthology, we should return to IGT's core foundation, i.e., the somatic marker hypothesis (SMH; Damasio, 1994). The SMH should also be reevaluated alongside other major approaches to the study of choice behavior under uncertainty, e.g., classical rational choice models, bounded rationality (Simon, 1955, 1956), prospect theory (Kahneman and Tversky, 1979), and modern neuroeconomics.

Despite their explanatory power, the traditional behavioral decision theories did not formally integrate emotional components into their human decision-making frameworks. A remarkable portion of emerging neuroeconomic research has focused on the impact of emotion on choice behavior. SMH and IGT could plausibly link traditional behavioral decision theory to emerging neuroeconomic research, highlighting the interplay between emotion and rationality in decision-making under uncertainty. A recent upsurge of interest in modeling emotion in artificial intelligence (AI) or social robotics systems could also be critically evaluated along with these studies: combining SMH and emotion theory within new-generation AI can potentially contribute much to current knowledge.

We therefore proposed a second Research Topic to serve these purposes: “*Iowa Gambling Task, Somatic Marker Hypothesis, and Neuroeconomics: Rationality and Emotion in Decision Under Uncertainty*.” To this end, we have anthologized 18 articles published in *Frontiers in Psychology* between 2018 and 2022. As with the first anthology, these articles encompass reviews, theoretical integration, clinical examinations, brain-imaging technology, and model construction. Like the first anthology, they center on the IGT and SMH while covering a range of additional issues as a springboard to further studies of the interaction between emotion and rationality in decision-making under uncertainty.

## Two Inseparable Entities: the SMH and the IGT

In traditional economic theory, rational economic decisions are defined as decisions aimed at maximizing monetary output (von Neumann and Morgenstern, 1947). Nevertheless, behavioral decision studies generally concur that choice behavior does not depend on the decision-maker's ability to calculate long-term overall gains and losses (Kahneman, 2003), fully refuting traditional models such as those of expected value or utility. In contrast to the neglect of emotion in these models, the SMH has stressed the facilitative role of emotion in decision-making under uncertainty (Damasio, 1994). For example, if decision-makers rely on skin conductance (SCR) as an index of somatic markers more than logic or logical reasoning (Bechara et al., 1997), they may increasingly predict long-term benefits, in a sense coming to see and choose good rather than bad decks. Conversely, the dysfunction of somatic marker systems can lead decision-makers to make risky and irrational choices.

For more than two decades, the revolutionary concept of SMH has framed studies of the impact of emotion on decision-making in dynamic-uncertain situations (Dunn et al., 2006). Interestingly, the IGT experiments undertaken by Bechara et al. (1994, 1997) remain a cornerstone of the concept's verifiability. The IGT itself was developed to evaluate realistic decision behavior in a dynamic-uncertain world. Bechara et al. (1994) designed the first dynamic-consecutive four-deck decision game, a significant departure from the descriptive games that preceded it. Participants did not know the outcome, gain-loss probability, or immediate gain-loss of the four decks. SMH shows that the choice patterns of healthy decision-makers can be predicted by eventual returns from the IGT.

As a complex gamble that simulates most types of gamble experience in dynamic-uncertain situations, the IGT thus offers an experimental platform for researchers to study the role of (unconscious) emotion in decision-making under uncertainty. The IGT requires participants to choose from four decks of cards marked A, B, C, and D, which each contain different proportions of “good” and “bad” cards. Whereas the average cost across more than ten trials from the “good” final outcome decks (A and B) will cost the decision-maker 250 USD, the same number of trials from the “bad” final outcome decks C and D will produce gains of 250 USD, on average. Decks C and D provide relatively small instant gains per trial but produce positive long-term consequences; A and B offer relatively large instant gains per trial but lead to negative final consequences in the long run (Bechara et al., 1994). This information is not shared with decision-makers, who therefore have no initial knowledge of the internal gamble structure and final outcomes. This lack of guidance on making the correct choices thus simulates the experience of a dynamic-uncertain world. Bechara et al. (1994) observed that from an initial position of ignorance, healthy participants gradually learned to distinguish between good and bad decks using emotional markers. On the contrary, participants with damage to the ventromedial prefrontal cortex (VMPFC) were powerless to suppress their tendencies to choose the bad decks, losing money consecutively and displaying myopic decision behavior.

However, subsequent findings have been inconsistent, with many IGT-related studies querying whether the IGT provides an adequate verification of SMH (Tomb et al., 2002; Maia and McClelland, 2004, 2005; Lin et al., 2007; Chiu et al., 2008, 2012). SMH assumes that healthy decision-makers will make positive selections that lead to positive final results during the IGT due to alert signals generated by somatic markers, with the reverse also holding for participants with VMPFC. However, additional mechanisms guiding choice behavior during the IGT have since been uncovered. Many researchers stress the importance of immediate gain-loss (as represented by the gain-loss frequency in the gamble) rather than expectations of long-term gains or losses. In these studies, “bad” deck B and “good” deck D appeared to be chosen because they contained higher probabilities of gain and lower probabilities of loss, irrespective of long-term outcomes. Remarkably, game outcomes indicated that SMH was comparable to SCR in healthy decision-makers and VMPFC patients alike.

In particular, the prominent deck B (PDB) phenomenon in which decision-makers prefer the higher frequency of gains but disadvantageous final result of deck B indicates the inability of some decision-makers to consider long-term outcomes/expectations during the IGT (for reviews, see Wilder et al., 1998; Lin et al., 2007; Chiu et al., 2008, 2012, 2018). These developments also resonate with conclusions from behavioral decision theory (Kahneman and Tversky, 1979; Kahneman, 2003) that decision-making under uncertainty is shortsighted, conflicting with the classical SMH understanding that decision-makers are primarily guided by foresight. If the frequency of gains or losses could explain participants' poor performance in the IGT, or if healthy participants exhibited patterns of shortsighted choice like those with VMPFC lesions (Caroselli et al., 2006), the SMH's basic assumptions would need to be revised. Additional research is therefore required to clarify the interaction between emotion and rationality in decision-making under uncertainty. Hopefully, such studies will highlight the relative strengths and weaknesses of the different approaches and point to a possible resolution of the core hypothesis.

## The Anthology

In the light of these considerations and following our second-round call for papers in 2018, this anthology was compiled from 18 published articles, each allocated to one of five categories, as summarized below.

### Reviews

Xu and Huang provide a mini-review of the evidence of IGT combined with SCRs, ERPs, and HR, while Lee et al. present cross-cultural evidence that the Prominent Deck B (PDB) phenomenon is widespread.

### Clinical Examinations

Na et al. consider the IGT and event-related potentials and demonstrate the net score was correlated with feedback-related negativity (FRN). Singh et al. provide evidence for changes in decision behavior in the IGT among people with left-hemispheric atrophy and hemispherectomy at the pre- and post-operational stages. Buelow and Brunell show that individuals who recalled painful social experiences preferred low-risk choices throughout the IGT, and there was no significant influence of narcissism in the balloon simulation risk task (BART), Columbia card task, Dice Game Task, or IGT. Martínez-García et al. show that eating disorder cases with more distorted body image made more disadvantageous or riskier decisions in the IGT. Gorzelańczyk et al. demonstrate that, compared with healthy control subjects, individuals with gambling addiction made riskier decisions in the IGT, whereas opiate addicts undergoing methadone treatment were less prone to risk-taking behaviors. Hengen and Alpers describe how they utilized the BART to evaluate the correlation between decision-making, social stress, and social anxiety. Xu et al. review research into IGT and schizophrenia and conducted empirical research showing that the PDB phenomenon applied to both schizophrenia and control groups, with the expected value rather less sensitive than gain-loss frequency in differentiating between the decision patterns of each group.

### Model Construction

Soshi et al. used empirical data and regression models to show that pre-specified state and anxiety traits forecast future choice behaviors differently. Harada explains how the Q-learning computation model was used to examine the influence of emotion and risk on divergent and convergent thinking. Merchán-Clavellino et al. integrated an unlimited-time version of the IGT, anticipatory skin conductance response, and probabilistic Prospect Valence Learning model to test the correlation of decision behavior and SMH.

### Theoretical Integration

In Singh et al.'s study, IGT data from three high, moderate, and low gender parity cultures (Germany, the United States, and India) was collected to test gender differences under the two phases (uncertainty and risk) of IGT. Using IGT and saliva testing, Singh shows that sex (testosterone) and stress (cortisol) hormones may be involved in regulating men's long-term decision-making in IGT. To target third question “(3) What kind of implicit or explicit learning/decision-making ability does the IGT actually test?”, Chiu et al., detailly reanalyzed the raw data of Maia and McClelland (2004) study. Chiu et al. found that in Bechara et al. (1997) and Maia and McClelland (2004) IGT studies with variant versions of the questionnaire, however, the IGT performance between both studies was partially different, this revealed both questionnaires might have the different suggestive effect for rational choice in the IGT. Notably, both datasets in Maia and McClelland (2004) study reveal that healthy decision-makers behave myopically, which is against the basic assumption of IGT (Bechara et al., 1994, 1997).

### Brain-Imaging Technology

Neo et al. describe research utilizing forced choice and economic tasks while observing right frontal goal conflict-specific EEG rhythms to detect changes in decision-making behavior under conflict, gain, and loss situations. Giustiniani et al. combined IGT and EEG to explore the relationship between motivation level and decision-making ability. Jäger et al. developed an iterative decision-making task in conjunction with fMRI studies and demonstrate that expected valence appears to be the best predictor of repetitive decision-making in the gambling task.

## Conclusion

The 18 articles summarized above are included in this second *Frontiers in Psychology* anthology of research into the interplay of rationality and emotion on decision behavior under dynamic-uncertain conditions. Overall, several tentative conclusions warranting further investigation can be drawn from the findings. First, some results support those of the previous anthology that the outcomes of the IGT may violate some of the core assumptions of the SMH. Second, the variable of gain and loss frequency in the game is instrumental in the decision-making process under uncertainty, irrespective of whether the uncertain choice settings are implicit or explicit. In this topic, we (Chiu et al.) re-raise the question of “what do IGT participants really know?”, which may remain controversial. However, we found that under uncertainty, most participants' decision-making was really behaved based on the gain-loss frequency. Third, the IGT continues to approximate real-life decision-making under uncertainty relatively realistic than the traditional, static, single-trial gambling task (Hastie and Dawes, 2010). This validity is reflected in its extension into clinical investigations and related brain-imaging studies of risk-taking, ensuring the IGT remains a critical experimental paradigm for future decision-making research (Hastie and Dawes, 2010), as this anthology shows. How best to reformulate the revolutionary scheme of the SMH in the light of increasingly diverse IGT results is a question that deserves our continuing attention (Chiu et al., 2018).

## Author Contributions

C-HL, Y-CC, and J-TH contributed to this article and finalized the draft after several rounds of detailed discussions. Y-CC and C-HL wrote the first draft. J-TH refined the manuscript. All authors contributed to the article and approved the submitted version.

## Funding

The work of the first author, C-HL, was kindly supported in part by the NSYSU-KMU Joint Research Project (#NSYSUKMU110-I001).

## Conflict of Interest

The authors declare that the research was conducted in the absence of any commercial or financial relationships that could be construed as a potential conflict of interest.

## Publisher's Note

All claims expressed in this article are solely those of the authors and do not necessarily represent those of their affiliated organizations, or those of the publisher, the editors and the reviewers. Any product that may be evaluated in this article, or claim that may be made by its manufacturer, is not guaranteed or endorsed by the publisher.
